# Lrp1 in osteoblasts controls osteoclast activity and protects against osteoporosis by limiting PDGF–RANKL signaling

**DOI:** 10.1038/s41413-017-0006-3

**Published:** 2018-02-26

**Authors:** Alexander Bartelt, Friederike Behler-Janbeck, F. Timo Beil, Till Koehne, Brigitte Müller, Tobias Schmidt, Markus Heine, Laura Ochs, Tayfun Yilmaz, Martin Dietrich, Jan P. Tuckermann, Michael Amling, Joachim Herz, Thorsten Schinke, Joerg Heeren, Andreas Niemeier

**Affiliations:** 1 Department of Orthopaedics, University Medical Center Hamburg-Eppendorf, Martinistr. 52, 20246 Hamburg, Germany; 2 Department of Biochemistry and Molecular Cell Biology, University Medical Center Hamburg-Eppendorf, Martinistr. 52, 20246 Hamburg, Germany; 3 Department of Osteology and Biomechanics, University Medical Center Hamburg-Eppendorf, Martinistr. 52, 20246 Hamburg, Germany; 4 Department of Orthodontics, University Medical Center Hamburg-Eppendorf, Martinistr. 52, 20246 Hamburg, Germany; 5 Department of Anatomy and Experimental Morphology, University Medical Center Hamburg-Eppendorf, Martinistr. 52, 20246 Hamburg, Germany; 6 Department of Molecular Genetics, University of Texas Southwestern Medical Center, Dallas, TX 75390 USA; 7 Institute for Comparative Molecular Endocrinology, University of Ulm, Ulm, Germany; 8 Present Address: Department of Genetics and Complex Diseases & Sabri Ülker Center, Harvard T.H. Chan School of Public Health, 665 Huntington Avenue, Boston, MA 02115 USA

## Abstract

Skeletal health relies on architectural integrity and sufficient bone mass, which are maintained through a tightly regulated equilibrium of bone resorption by osteoclasts and bone formation by osteoblasts. Genetic studies have linked the gene coding for low-density lipoprotein receptor-related protein1 (Lrp1) to bone traits but whether these associations are based on a causal molecular relationship is unknown. Here, we show that Lrp1 in osteoblasts is a novel regulator of osteoclast activity and bone mass. Mice lacking Lrp1 specifically in the osteoblast lineage displayed normal osteoblast function but severe osteoporosis due to highly increased osteoclast numbers and bone resorption. Osteoblast Lrp1 limited receptor activator of NF-κB ligand (RANKL) expression in vivo and in vitro through attenuation of platelet-derived growth factor (PDGF-BB) signaling. In co-culture, Lrp1-deficient osteoblasts stimulated osteoclastogenesis in a PDGFRβ-dependent manner and in vivo treatment with the PDGFR tyrosine kinase inhibitor imatinib mesylate limited RANKL production and led to complete remission of the osteoporotic phenotype. These results identify osteoblast Lrp1 as a key regulator of osteoblast-to-osteoclast communication and bone mass through a PDGF–RANKL signaling axis in osteoblasts and open perspectives to further explore the potential of PDGF signaling inhibitors in counteracting bone loss as well as to evaluate the importance of functional *LRP1* gene variants in the control of bone mass in humans.

## Osteoporosis: A novel path to strength

Maintaining strong bones critically depends on a receptor (Lrp1) for low-density lipoprotein. Bones are continually remodeled, with osteoblast cells adding new bone and osteoclast cells resorbing old bone. Imbalanced growth and resorption can lead to osteoporosis. Genetic studies had previously linked Lrp1 to bone health, but the nature of the link remained unknown. Andreas Niemeier at the University Medical Center Hamburg-Eppendorf in Germany and co-workers used model mice whose osteoblasts lacked Lrp1 to investigate how the receptor is involved in bone turnover. Lrp-1-deficient mice showed severe osteoporosis. They also showed high numbers of osteoclasts but normal numbers of osteoblasts, indicating that lack of the receptor caused increased bone resorption. Treatment of the mice with a drug related to Lrp1 restored bone strength. These results may help to identify new treatments for bone loss.

## Introduction

In osteoporosis and tumor-associated osteolysis, bone resorption prevails over bone formation, leading to low bone mass, inferior bone architecture, and increased fracture risk^1,2^. RANKL is an essential osteoclast differentiation and activation factor synthesized by the cells of the osteoblast lineage and its action is balanced by binding to its soluble decoy receptor osteoprotegerin (OPG)^3,4^. In addition, RANKL action contributes to tumor development^5,6^ and both postmenopausal osteoporosis and tumor-associated bone loss can be effectively treated by RANKL antibody therapy^1,2^. Identifying new regulators of RANKL action can open additional therapeutic avenues for treating low-bone mass phenotypes in several clinical settings. Genetic studies have linked the gene coding for low-density lipoprotein receptor-related protein1 (Lrp1) to bone traits^7,8^ but whether these associations are based on a causal molecular relationship is unknown. Lrp1 is a multi-functional member of the low-density lipoprotein (LDL) receptor (LDLR) family^9^ and mice systemically deficient in Lrp1 are not viable^10^. Using conditional gene targeting, several functions of Lrp1 have been unraveled in mice, amongst these an endocytic role in the clearance of plasma remnant lipoproteins^11^. Through interaction with other receptors at the cell surface, Lrp1 can also modulate cellular signaling pathways. As such, it has been described that by modulation of platelet-derived growth factor (PDGF) receptor-β (PDGFRβ) stability and signaling, Lrp1 has an atheroprotective effect in the vessel wall^12^. With regard to the skeletal system, we have shown that Lrp1 acts as a lipoprotein receptor that is highly expressed in human osteoblasts^13,14^ and recent in vitro studies suggested a role of Lrp1 in chondrocyte differentiation^15^. Despite these reports, there is no in vivo evidence, e.g., from transgenic animal models, for a definitive significance of Lrp1 in skeletal biology. Here, we analyze the function of Lrp1 in osteoblasts in vivo by genetic means and demonstrate that osteoblast Lrp1 protects against osteoporosis by limiting a novel PDGF–RANKL signaling axis in osteoblast-to-osteoclast communication.

## Results

### Generation of a conditional transgenic mouse model for studying osteoblast Lrp1

**Fig. 1:**
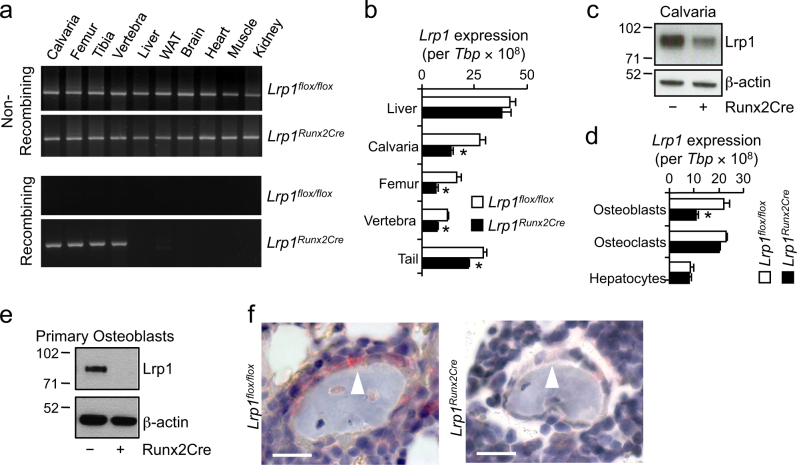
Osteoblast-specific disruption of Lrp 1. **a** PCR detection of cre-mediated recombination of floxed *Lrp1* alleles in *Lrp1*^*Runx2Cre*^ mice and *Lrp1*^*flox/flox*^ controls. WAT white adipose tissue. **b** Tissue *Lrp1* mRNA levels quantified by real-time PCR. **c** Immunoblot of calvarial Lrp1 protein. **d** Primary cells *Lrp1* mRNA levels quantified by real-time PCR. **e** Primary osteoblast Lrp1 immunoblot as well as **f** in vivo immunohistochemistry of Lrp1 protein in sections from distal femur trabecular bone of *Lrp1*^*Runx2Cre*^ mice and *Lrp1*^*flox/flox*^ controls. Scale bar, 25 µm. Means ± s.e.m. *n* > 5 per group (*P < 0.05 as determined by two-tailed, unpaired *T*-test)

To test our hypothesis that osteoblast Lrp1 is physiologically relevant, we generated mice carrying conditional (floxed) *Lrp1* alleles^16^ and osteoblast-specific expression of *Cre* recombinase under control of the *Runx2*-promoter,^17^ hereafter referred to as *Lrp1*^*Runx2Cre*^ animals. Littermates without Cre expression served as controls (*Lrp1*^*flox/flox*^). We verified the specificity of the conditional knock-out approach by detecting non-recombining and recombining parts of the *Lrp1* alleles in genomic DNA extracted from various tissues by polymerase chain reaction (PCR) (Fig. 1a): only in osteoblast-containing bone tissues of *Lrp1*^*Runx2Cre*^ animals the recombined null allele was detected. Quantification of *Lrp1* mRNA levels in a variety of bone tissues by quantitative real-time PCR displayed that *Lrp1* expression was reduced by 40-60% compared to *Lrp1*^*flox/flox*^ controls (Fig. 1b). In the liver, a major site of Lrp1 function,^11^ *Lrp1* mRNA levels were unchanged (Fig. 1b). Reduced *Lrp1* mRNA levels in calvaria were translated into less Lrp1 protein (Fig. 1c). Furthermore, there was a strong reduction of *Lrp1* expression in primary osteoblasts whereas in primary osteoclasts and in primary hepatocytes, *Lrp1* mRNA levels remained unaltered (Fig. 1d). This reduction in mRNA expression resulted in a strong reduction of Lrp1 protein levels in primary osteoblasts (Fig. 1e). Immunohistochemistry staining of trabecular bone revealed a specific deletion of Lrp1 protein in osteoblasts in vivo (Fig. 1f and Supplementary Fig. 1).

### Lipoprotein metabolism in mice lacking osteoblast Lrp1

Based on our previous work on the role of osteoblast Lrp1 for internalizing triglyceride-rich lipoproteins and vitamin K delivery to osteoblasts in vitro, we tested whether *Lrp1*^*Runx2Cre*^ mice display abnormalities in their lipoprotein metabolism. There was no detectable contribution of osteoblast Lrp1 to systemic lipoprotein clearance as there was no change in either fasting or postprandial lipoprotein concentrations upon deletion of osteoblast Lrp1 (Supplementary Fig. 2a-d). We also tested whether *Lrp1*^*Runx2Cre*^ mice show impaired delivery of postprandial lipoproteins to bone locally using radiolabeled tracers for fatty acids as well as entire lipoproteins. Interestingly, loss of Lrp1 in osteoblasts did not reduce the capacity of bone tissues to sequester lipids from the circulation (Supplementary Fig. 2e-f).

### Osteoporosis in mice lacking osteoblast Lrp1

**Fig. 2:**
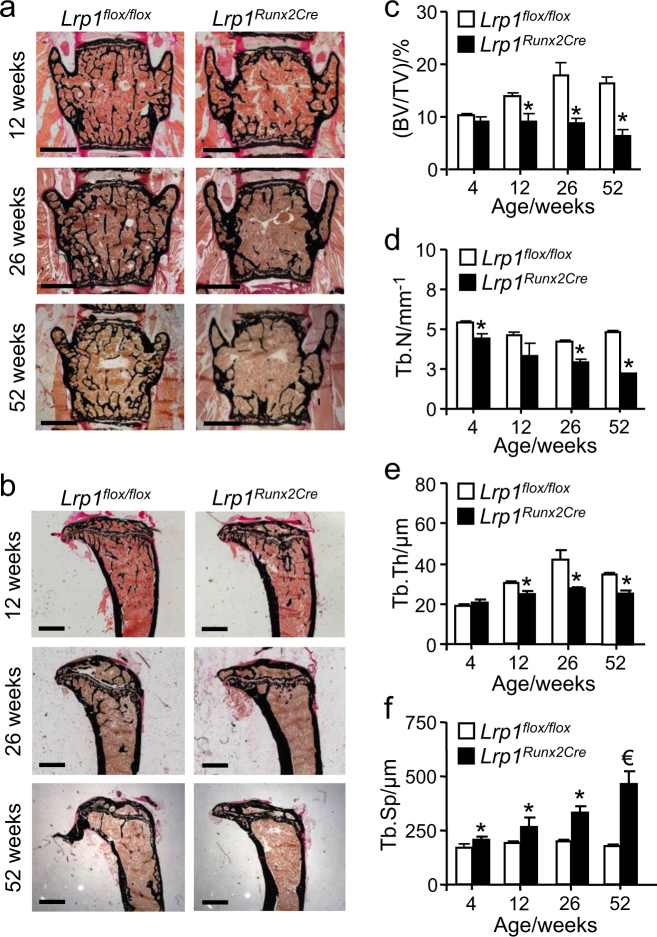
Loss of Lrp1 in osteoblasts results in an age-dependent osteoporotic phenotype. **a** Histology of vertebra and **b** tibia in female *Lrp1*^*Runx2Cre*^ mice compared to *Lrp1*^*flox/flox*^ controls. Scale bar, 1 mm. **c** Histomorphometric analysis of lumbar vertebra with bone volume per total volume (BT/TV), **d** trabecular number (TbN), **e** trabecular thickness (TbTh), **f** trabecular spacing (TbSp). Means ± s.e.m. *n* > 5 per group (*€*P* < 0.05 as determined by two-tailed, unpaired *T*-test)

**Fig. 3:**
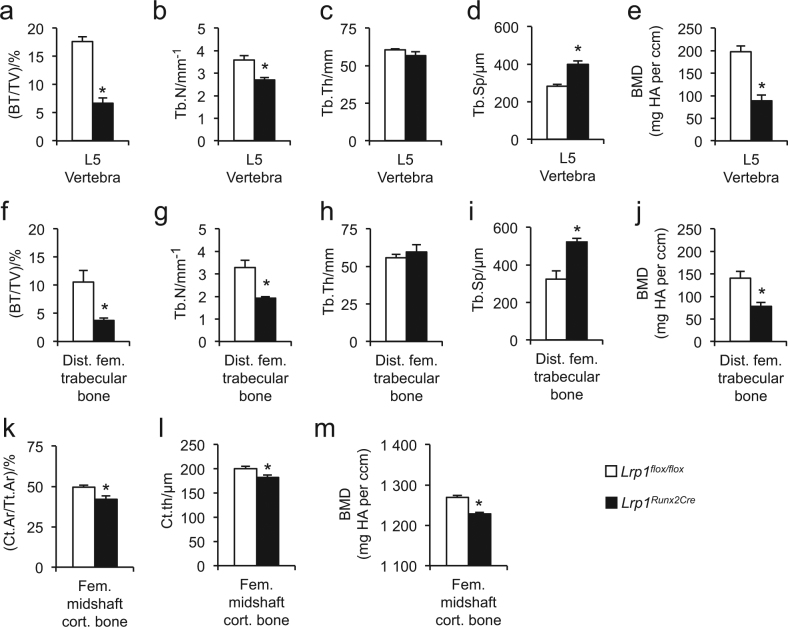
Loss of Lrp1 in osteoblasts results in deteriorated bone architecture. µCT analysis of L5 vertebra from 26-week-old female *Lrp1*^*Runx2Cre*^ mice compared to *Lrp1*^*flox/flox*^ controls for **a** bone volume per total volume (BT/TV), **b** trabecular number (TbN), **c** trabecular thickness (TbTh), **d** trabecular spacing (TbSp), and **e** bone mineral density (BMD), and for distal femoral trabecular bone (**f–j**) as well as femoral midshaft cortical bone (**k**) cortical area per total area (Ct.Ar per Tt.Ar), (**l**) cortical thickness (Ct.Th), and (**m**) BMD. Means ± s.e.m. *n* = 4–6 per group (*P < 0.05 as determined by two-tailed, unpaired *T*-test)

In contrast to the unchanged lipoprotein phenotype, we observed a remarkable age-dependent severe osteoporosis. Undecalcified histological bone sections of *Lrp1*^*Runx2Cre*^ mice revealed a low-bone mass phenotype at 12 weeks of age and progressively thereafter in lumbar vertebrae (Fig. 2a) as well as in tibiae (Fig. 2b). Histomorphometric analyses of lumbar vertebrae revealed that *Lrp1*^*Runx2Cre*^ mice were characterized by low trabecular bone mass with deteriorated bone architecture (Fig. 2c-f), which became more pronounced over time and resulted in severe osteoporosis at 12 months of age. Severe loss of trabecular and cortical bone was also observed by µCT analysis of the femur and lumbar spine. In the distal femur as well as in lumbar vertebrae of *Lrp1*^*Runx2Cre*^ mice, the osteoporotic phenotype was confirmed (Fig. 3a-j). In addition, femoral midshaft cortical thicknesses as well as cortical bone mineral density were reduced in *Lrp1*^*Runx2Cre*^ mice (Fig. 3k–m). Taken together, histology and µCT demonstrated a severe, age-dependent bone loss in trabecular and cortical compartments of the appendicular and axial skeleton.

### Osteoblast Lrp1 controls osteoclastogenesis

**Fig. 4:**
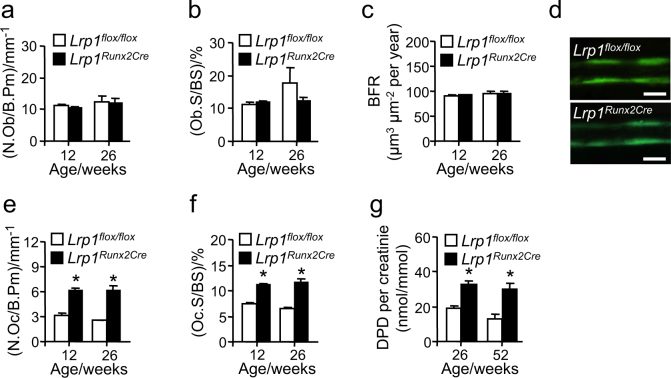
Loss of Lrp1 in osteoblasts results in increased osteoclastogenesis in vivo. Histomorphometric analysis of lumbar vertebra for **a** osteoblast number (ObN) per bone perimeter (BPm), **b** osteoblast surface (ObS) per bone surface (BS), **c** bone formation rate (BFR), and **d** BFR assessed by double-calcein labeling. **e** Osteoclast number (OcN) per BPm and **f** osteoclast surface (OcS) per BS as well as **g** urinary bone resorption product deoxypyridinoline (DPD) per creatinine in female *Lrp1*^Runx2Cre^ mice compared to *Lrp1*^*flox/flox*^ controls. Means ± s.e.m., *n* > 5 per group. (*P < 0.05 as determined by two-tailed, unpaired *T*-test)

To further delineate the bone phenotype of *Lrp1*^*Runx2Cre*^ mice, osteoblast and osteoclasts cell counts were determined by histomorphometry. Neither osteoblast number (Fig. 4a) nor surface (Fig. 4b) were altered in *Lrp1*^*Runx2Cre*^ animals compared to controls. In line, the dynamic bone formation rate as assessed by double-calcein labeling remained unchanged (Fig. 4c, d). Also in vitro, osteoblast differentiation was not affected by loss of *Lrp1* (Supplementary Fig. 3). In stark contrast, we found strongly increased osteoclast numbers (Fig. 4e) as well as osteoclast surface (Fig. 4f). Correspondingly, concentrations of the bone resorption product deoxypyridinoline in urine were highly increased in *Lrp1*^*Runx2Cre*^ animals (Fig. 4g). In vitro, however, isolated primary osteoclasts from *Lrp1*^*Runx2Cre*^ mice and controls did not show differences in differentiation and activation (Supplementary Fig. 4). Furthermore, mice lacking Lrp1 specifically in osteoclasts displayed normal bone mass compared to controls (Supplementary Tab. 1), confirming that *Lrp1* expression in osteoblasts is pivotal to controlling osteoclastogenesis.

### Osteoblast Lrp1 limits RANKL production

**Fig. 5:**
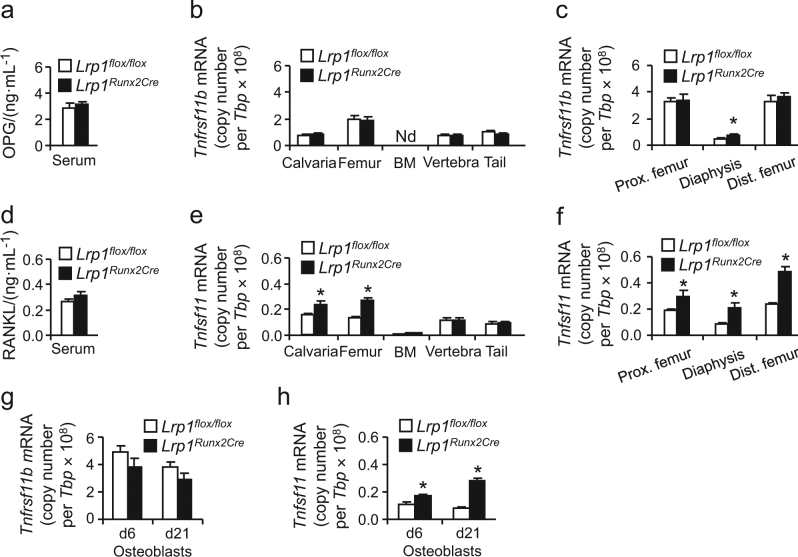
Loss of Lrp1 in osteoblasts results in increased RANKL expression in vivo and in vitro. **a** Serum OPG levels. **b**, **c** In vivo bone expression of *Tnfrsf11b* (encoding OPG) quantified by real-time PCR. Nd not detectable. **d** Serum RANKL levels. **e** In vivo bone expression of *Tnfsf11* (encoding RANKL) and (**f**) in parts of the femur quantified by real-time PCR. Means ± s.e.m., *n* = 6 per group. **g** *Tnfrsf11b* expression and **h** *Tnfsf11* expression in primary calvarial osteoblasts at days 6 and 21 in differentiation, two independent experiment in triplicates; **a**–**h**, *P < 0.05 as determined by two-tailed, unpaired *T*-test

In order to gain deeper insight into the mechanisms underlying the osteoporotic phenotype in *Lrp1*^*Runx2Cre*^ mice, we analyzed the standard serum parameters of bone turnover (Supplementary Tab. 2): neither alkaline phosphatase, parathyroid hormone, vitamin D, calcium, phosphate nor glucose levels were altered (Supplementary Tab. 2). Osteocalcin levels were slightly higher in *Lrp1*^*Runx2Cre*^ mice (Supplementary Tab. 2). Given the key role of the OPG–RANKL system in coupling bone resorption to formation and osteoblast-to-osteoclast communication,^18^ we measured serum and bone expression levels of OPG and RANKL. We found serum OPG levels (Fig. 5a) as well as the expression of gene coding for OPG, *Tnfrsf11b*, in bone tissues to be unchanged in *Lrp1*^*Runx2Cre*^ mice compared to controls except for a slight upregulation in the femoral diaphysis of *Lrp1*^*Runx2Cre*^ animals (Fig. 5b, c). Surprisingly, also serum RANKL concentrations were unchanged in *Lrp1*^*Runx2Cre*^ mice compared to controls (Fig. 5d). As RANKL is a trans-membrane protein that can act locally without systemic changes in the circulation,^18^ we also measured mRNA expression levels of the gene coding for RANKL, *Tnfsf11*, in bone tissues of *Lrp1*^*Runx2Cre*^ mice and controls: we found a marked increase in *Tnfsf11* expression in bone tissues, most pronounced in calvaria and femur (Fig. 5e, f). While in primary isolated osteoblasts *Tnfrsf11b* remained unchanged (Fig. 5g), the increased *Tnfsf11* expression in vivo was mimicked in vitro in *Lrp1*^*Runx2Cre*^ osteoblasts early and late in differentiation (Fig. 5h). In summary, these results indicate that Lrp1 deletion in osteoblasts leads to locally increased RANKL levels within bone, explaining both increased osteoclast numbers and activity as well as the marked loss of bone mass. While we systematically and comprehensively studied female mice for bone analyses, we confirmed that an increase in *Tnfsf11* (RANKL) expression was also observed in bones of male mice lacking Lrp1 in osteoblasts (Supplementary Fig. 5).

### PDGF signaling links osteoblast Lrp1 to RANKL production in cultured cells

**Fig. 6:**
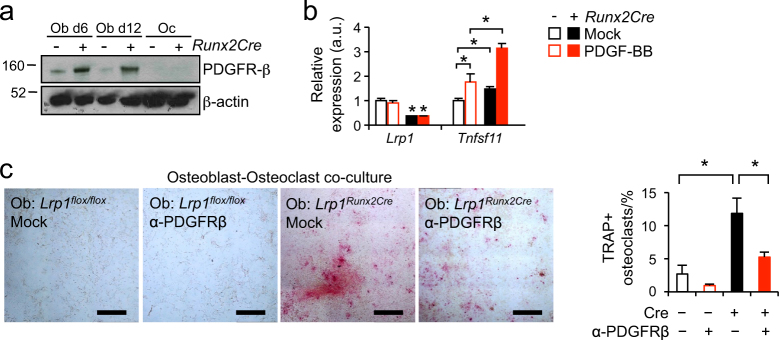
Lrp1 modulates RANKL expression and osteoclastogenesis by limiting PDGF signaling. **a** Immunoblot detection of PDGFRβ in primary calvarial osteoblasts at days 6 and 12 of differentiation. Ob osteoblast, Oc osteoclast. **b** *Lrp1* and *Tnfsf11* (encoding RANKL) expression levels quantified by real-time PCR in control and PDGF-BB-stimulated primary calvarial osteoblasts. **c** Representative images and quantification of osteoblast–osteoclast co-culture experiments. Co-culture of primary wild-type osteoclasts with primary Lrp1-deficient osteoblasts compared to co-culture with primary wild-type osteoblasts increased osteoclastogenesis, which was blocked by an anti-PDGFRβ antibody (Two independent experiment in triplicates). Bar: 500 μmol·L^-1^ (*P < 0.05 as determined by 2-way ANOVA followed by Tukey's Test on data from two independent experiments in triplicates)

In search of the molecular mechanism upstream of the induction of RANKL expression, we hypothesized that PDGF signaling may be involved as Lrp1 is pivotal to the control of cellular PDGFRβ abundance and activity in other organs and cell types.^19–21^. PDGF signaling in turn has been indirectly implicated in osteoclastogenesis and osteoclast function in vitro^22,23^. We hypothesized that increased PDGF signaling in Lrp1-deficient osteoblasts might be responsible for the increased RANKL levels in bones of *Lrp1*^*Runx2Cre*^ mice. Indeed, we found increased protein expression of PDGFRβ in primary osteoblasts from *Lrp1*^*Runx2Cre*^ mice compared to control cells (Fig. 6a) and, therefore, asked whether PDGF signaling might be regulating *Tnfsf11* expression in osteoblasts. After short-term stimulation with PDGF-BB in control cells, *Tnfsf11* expression was increased about 2-fold whereas this induction was significantly higher in cells isolated from *Lrp1*^*Runx2Cre*^ mice (>3-fold, *P* < 0.05) (Fig. 6b). As loss of Lrp1 in osteoblasts resulted in increased PDGF signaling and subsequently increased RANKL levels, we hypothesized that co-culture of primary osteoblasts isolated from *Lrp1*^*Runx2Cre*^ mice with wild-type osteoclast precursors should increase osteoclastogenesis. We found that co-culture of Lrp1-deficient osteoblasts displayed elevated TRAP staining in contrast to wild type osteoblast, indicating stimulated osteoclastogenesis in the absence of Lrp1 (Fig. 6c). The addition of an antibody against PDGFRβ that blocks PDGF signaling markedly reduced osteoclastogenesis in the co-culture system (Fig. 6c). These results indicate that PDGF signaling is an important mediator of the effects of osteoblast Lrp1 on osteoclasts in vitro.

### Treatment of mice lacking osteoblast Lrp1 with Imatinib leads to remission of osteoporosis

**Fig. 7:**
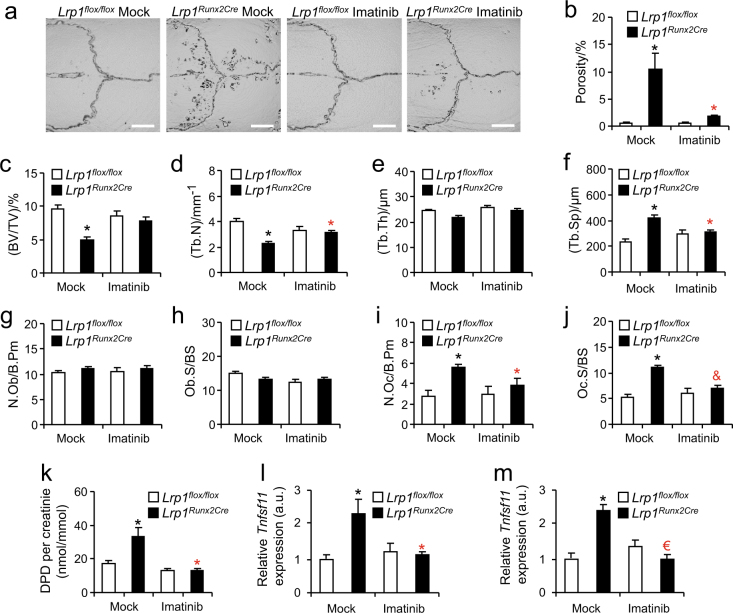
An Lrp1–PDGF–RANKL axis in osteoblasts controls osteoporosis. **a**, **b** Female *Lrp1*^*Runx2Cre*^ mice and *Lrp1*^*flox/flox*^ controls were fed an Imatinib-containing or mock diet from the age of 4 to 26 weeks. µCT analysis of calvaria for imaging and quantification of osteoclast resorption pits. Scale bar, 2 mm. **c–f** Histomorphometric analysis of lumbar vertebrae. **g** Osteoblast number, **h** osteoblast surface, **i** osteoclast number, **j** osteoclast surface, and **k** urinary DPD bone resorption levels. **l** Real-time PCR quantification of *Tnfsf11* (encoding RANKL) in femoral diaphysis and **m** distal femur. Means ± s.e.m., *n* > 5 per group. Statistically significant differences between genotypes are indicated in black symbols and between diets in red symbols (*&€ *P* < 0.05 as determined by 2-way ANOVA followed by Tukey's Test)

Seeking to corroborate this concept in vivo, we hypothesized that treatment of *Lrp1*^*Runx2Cre*^ mice with the PDGF signaling inhibitor Imatinib mesylate should normalize the aberrantly increased *Tnfsf11* expression in Lrp1-deficient osteoblasts, thus correcting the low-bone mass phenotype. In fact, we found that Imatinib treatment from 4 to 26 weeks of age strongly decreased the porosity as well as reduced the number of osteoclast resorption pits in calvaria (Fig. 7a, b and Supplementary Fig. 6) of *Lrp1*^*Runx2Cre*^ mice, indicating blunted osteoclast activity in *Lrp1*^*Runx2Cre*^ mice while in *Lrp1*^*flox/flox*^ controls, Imatinib had no effect compared to the mock-treated group (Fig. 7a, b). In line, histomorphometric analysis of lumbar vertebrae indicated that Imatinib treatment resulted in increased trabecular bone mass and improved bone architecture in *Lrp1*^*Runx2Cre*^ mice whereas in *Lrp1*^*flox/flox*^ control animals, Imatinib had no effect (Fig. 7c-f). Furthermore, while there was no effect on osteoblasts (Fig. 7g, h), osteoclast numbers and surface in *Lrp1*^*Runx2Cre*^ animals fed with Imatinib returned to levels found in *Lrp1*^*flox/flox*^ controls (Fig. 7i, j). This was also reflected by normal urinary DPD/creatinine levels in the Imatinib-treated *Lrp1*^*Runx2Cre*^ animals (Fig. 7k). This phenotypic normalization can be explained by a suppression of *Tnfsf11* expression, as exemplified in the diaphysis (Fig. 7l) and distal femur (Fig. 7m) of *Lrp1*^*Runx2Cre*^ mice. Taken together, these data support the concept that loss of Lrp1 in osteoblasts leads to increased osteoblast PDGF signaling, which in turn stimulates *Tnfsf11* expression and thereby increases osteoclast numbers and activity, resulting in severe osteoporosis of all bone compartments in mice.

## Discussion

Here, we identify osteoblast Lrp1 as a key regulator of cortical and trabecular bone mass in mice, uncovering a novel control mechanism of the osteoblast-to-osteoclast communication via a previously unrecognized Lrp1–PDGF–RANKL axis in osteoblasts. These findings now call for a systematic analysis of LRP1 variants and their respective allele frequencies in human low-bone mass disorders and also open perspectives to further explore the potential of tyrosine kinase inhibitors in counteracting bone loss.

The current study contributes to the general understanding of skeletal biology by several ways and opens clinically relevant perspectives. As the LDLR-related proteins Lrp4, 5, and 6 are clinically targeted critical regulators of bone mass,^24^ the discoveries on Lrp1 here may bear potential for new therapeutic options. Since Lrp1 displays partially overlapping but distinct ligand-binding properties compared to the other members of the LDLR family,^9^ the targeting of Lrp1-mediated signaling in bone cells should be considered in the context of the interaction with other LDLR family members. This is particularly relevant as LDLR family members can compensate for some of each other’s functions and a combined therapy might therefore be more efficient. Of interest in this context, the LDLR and Lrp1 are cooperating as well as compensating for each other in their function as hepatic lipoprotein clearance receptors genetic deletion models,^11,25^ which might explain the unchanged lipoprotein uptake into bone in osteoblast Lrp1-deficient mice in the current study. Of note, global LDLR deficiency in mice is associated with a high-bone mass phenotype, decreased osteoclastogenesis, and osteoclast function,^26^ further underlining the principal relevance of this receptor family for bone cell function. However, the particular cell-type specific interaction of the LDLR and Lrp1 as well as between Lrp1 and Lrp 4, 5, and 6 will need to be explored by bone-cell specific conditional targeting of each receptor.

While in vitro evidence for a role of PDGF signaling in bone cells has been described earlier, in particular by increasing osteoclastogenesis via stromal cell-dependent mechanisms^23,27^ and by increasing OPG production through inhibition of PDGFRβ signaling, our present findings reveal for the first time that Lrp1-mediated modulation of PDGFRβ signaling regulates osteoblast-to-osteoclast communication via RANKL in vitro and in vivo. The current in vivo demonstration of an Lrp1–PDGF–RANKL signaling axis in the regulation of bone mass throughout the entire skeleton offers opportunities to further explore clinical conditions, in which manipulation of PDGF signaling might be beneficial for the preservation of bone mass. Albeit speculative at present, we propose that the molecular mechanism linking PDGF, Lrp1, and RANKL, all of which have been independently implicated in various tumors, may help to also understand the ways of reducing tumor-associated osteolysis.

There is now abundant evidence for a general and evolutionarily ancient role of several LDLR receptor family members in the regulation of most, if not all, of the classic and fundamental signaling pathways that govern development, cell growth and differentiation, signaling, and metabolism, such as tyrosine, serine and threonine kinases, ion channels, Wnt, Bmp, Shh, Tgfβ, etc.^9,24^. Therefore, although we provide evidence in vivo and in vitro that primarily PDGF-BB PDGFRβ signaling is mediating the effects of osteoblast Lrp1 deficiency to the control of bone mass via osteoclasts, based on the variety of ligands binding to Lrp1 and the number of co-receptors that Lrp1 interacts with,^9^ we do not rule out that other signaling pathways might also be modulated by Lrp1 in osteoblasts. It is important to point out that in contrast to osteoblasts, Lrp1 in osteoclasts is dispensable, as we do not find any abnormal skeletal alterations in mice lacking Lrp1 specifically in osteoclasts. This implies that targeting Lrp1-mediated pathways in the osteoblast lineage may represent a novel and safe avenue toward the treatment of bone loss.

## Methods

### Mice and treatments

The Animal Welfare Officers of University Medical Center Hamburg-Eppendorf and Behörde für Gesundheit und Verbraucherschutz Hamburg, Germany, approved all experimental procedures. *Lrp1*^*flox/flox*^ mice as well as mice carrying Cre under control of the *Runx2* promoter were described previously^11, 16,17^. Mice (C57BL/6 background) were bred and housed in the animal facility of University Medical Center Hamburg-Eppendorf at 22 °C with ad libitum access to standard laboratory chow diet (Ssniff) or chow diet supplemented with 100 mg/kg diet Imatinib (Novartis, Imatinib diet and control diet custom-made by Ssniff). Age- and sex-matched littermates were randomly used for the study of adult mice. Twelve-week-old female mice were studied unless indicated otherwise. Age-matched litters were used to achieve statistical power and sufficient sample size (at least *n* = 5 as indicated in the figures). For bone formation rate assessment, we used a standard calcein 7-day interval double-labeling method as described previously^28^. Standardized necropsies were performed after 4 h fasting around noon. Mice were anesthetized with a lethal dose of Ketamine/Xylazine and blood was withdrawn by cardiac puncture. Organs were harvested and immediately conserved in TRIzol (Invitrogen), formalin, or snap-frozen in liquid nitrogen and stored at −80 °C. Skeletons were mounted, fixed overnight in 3.7% formalin, and stored in 80% ethanol. X-ray radiography was performed using an X-ray cabinet (Faxitron).

### Primary cell preparation and culture

Primary osteoblasts were isolated by sequential collagenase Ia (Sigma) digestion of calvaria from 3-day-old mice (male and female) as described previously^29^. Osteoblast differentiation was induced at 80% confluence in αMEM (Sigma) containing 10% fetal bovine serum (Perbio), 50 µg·mL^-1^ ascorbic acid, and 10 mmol·L^-1^ β-glycerophosphate (Sigma). At indicated time points, cells were fixed in 4% PFA (Merck) or directly harvested in TRIzol for RNA isolation (see below). For PDGF-bb stimulation, cells were differentiated to day 6, starved for 24 h in DMEM (Invitrogen) containing 0.01% fetal bovine serum, and stimulated for 6 h with 5 or 20 ng·mL^-1^ PDGF-BB (Peprotech) in starvation medium. At indicated time points, cells were harvested in TRIzol for RNA isolation (see below). For primary osteoclasts, femoral bone marrow of 12-week-old male mice was harvested by centrifugation (10 s at 8400×*g*) and whole marrow cells were seeded at a concentration of 5 × 10^6^ per mL in αMEM (Sigma) in 6-well plates. Differentiation was induced by adding macrophage colony-stimulating factor (Peprotech) to 20 ng·mL^-1^ and RANKL to 40 ng·mL^-1^ (Peprotech) for 3 days. Cells were directly harvested in TRIzol for RNA isolation (see below) or fixed for 5 min with ice-cold methanol (Merck) for immediate tartrate-resistant acid phosphatase (TRAP) staining. Cell were washed with water, dried for 2 min at room temperature, and developed with freshly prepared staining solution (40 mmol·L^-1^ sodium acetate, Merck; 10 mmol·L^-1^ sodium tartrate, Merck; 0.1 mg·mL^-1^ naphtol AS-MX phosphate, Sigma; 0.6 mg·mL^-1^ Fast Red Violet LB salt, Sigma; 1% V/V N,N-Dimethylformamide, Sigma). TRAP-positive giant multinucleated cells (GMNCs) were counted using a threshold method with Image J^28^. Primary hepatocytes were prepared as described^30^. Briefly, hepatocytes were seeded in DMEM containing 10% FCS (Invitrogen) to a density of 200 000 cells per well in collagen-coated 12-well plates (Nunc) and harvested in TRIzol 24 h after seeding. At least 2–3 independent experiments were performed in duplicates or triplicates.

### Serum and urine parameters

Triglycerides and cholesterol were determined using commercial kits (Roche) that were adapted to microtiter plates. For fast performance liquid chromatography (FPLC), pooled plasma was separated using S6-superose columns (GE Healthcare) and lipid levels were analyzed in each fraction as described above. Oral fat tolerance tests were performed as described previously^31^. Alkaline phosphatase was determined using the NPP method (Sigma). Osteocalcin was analyzed by an immunoradiometric assay (Immutopics) and undercarboxylated osteocalcin was determined after hydroxylapatite wash and subsequent ELISA (immuntopics) detection as described previously^14^. OPG and RANKL (R&D), DPD and creatinine (Quidel), parathyroid hormone (immutopics), and Vitamin Ds (BlueGene) were determined by ELISA according to manufacturer’s instructions. Blood glucose levels were measured using AccuCheck Aviva sticks (Roche).

### Gene expression analysis

Cre-mediated recombination of floxed *Lrp1* alleles was detected in genomic DNA by PCR as described previously:^32^ non-recombining as well as recombining parts of the floxed *Lrp1* alleles were amplified. Cells or tissues in TRIzol (Invitrogen) were disrupted using TissueLyser (Qiagen). Total RNA was isolated using NucleoSpin RNA II kit (Macherey & Nagel). Complementary DNA was synthesized using SuperScript® III Reverse Transcriptase (Invitrogen). Quantitative real-time PCR was performed in quadruplicates on a 7900HT sequence detector (Applied Biosystems) using TaqMan Assay-on-Demand primers supplied by Applied Biosystems. Gene of interest cycle thresholds (Cts) were normalized to *TATA-box binding protein* (*Tbp*) house keeper levels by the ΔΔCt method and displayed as relative copies per *Tbp* or relative expression normalized to experimental control groups.

### Bone histomorphometry

For comparative quantitative histomorphometry, undecalcified lumbar spines (L2–L4) were embedded in methylmethacrylate after dehydration and 5-µm sections were cut in the frontal plane on a rotation microtome (Cut 4060E, MicroTech). Sections were stained with von Kossa/van Gieson or Toluidine Blue. Analysis of bone volume (BV/TV), trabecular thickness (Tb.Th), trabecular number (Tb.N), trabecular separation (Tb.Sp), osteoblast number per bone perimeter (N.Ob/B.Pm), osteoblast surface per bone surface (Ob.S/BS), osteoclast number per bone perimeter (N.Oc/B.Pm), osteoclast surface per bone surface (Oc.S/BS), and trabecular bone formation rate (BFR) was performed on L3 and L4 according to standardized protocols of the American Society for Bone and Mineral Research^33^ using the Osteomeasure histomorphometry system (Osteometrix) in a blinded fashion only knowing animal numbers but not genotypes or treatments.

### Immunoblot analysis

Primary cells were harvested in radioimmunosorbent assay (RIPA) buffer, tissue samples were snap-frozen in liquid nitrogen. Cells or tissues were disrupted using a TissueLyser (Qiagen) in RIPA buffer. Equal amounts of total cell protein were separated using NuPAGE® Novex 4%-12% Bis-Tris Mini Gels with MES SDS running buffer (Invitrogen) at 4 °C, transferred to Protran Nitrocellulose Blotting Membrane (GE Health Care Life Sciences) overnight at 4 °C, blocked with milk powder at room temperature (RT), washed 3× with Tris-buffered saline with 0.1% w/v Tween (TBST) at RT, incubated with primary antibodies (Lrp1: polyclonal LRP1-377, provided by J.Her.; PDGFRβ: Millipore 06-498; β-actin: Sigma, clone AC-15, 06-A5441) in TBST with 10% w/v bovine serum albumin (Sigma) overnight at 4 °C, washed 3× with TBST (RT), incubated with secondary horseradish peroxidase-labeled antibodies (Jackson ImmunoResearch) in TBST for 90 min at RT, washed 3× with TBST, and finally 1× with PBS. Immunoblots were developed with enhanced chemiluminescence (GE Health Care Life Sciences).

### Bone immunohistochemistry

Knees were fixed in 3.7% formalin as described above and embedded in paraffin. Five-μm-thick sections were cut, dewaxed, microwaved in Target Retrieval Solution (DAKO) for 2 × 4 min and cooled down to RT for 40 min. After washing with TBS, slides were blocked by incubating sections in 10% normal swine serum (DAKO) for 30 min at RT. Then, the slides were incubated with anti-Lrp1 antibody (polyclonal LRP1-377, provided by J.Her.) or rabbit control antibody at a dilution of 1 µg·mL^-1^ for 60 min at RT, followed by a biotinylated rabbit anti-mouse antibody (DakoCytomation) at a dilution of 1:200 for 30 min. Hereafter, the slides were washed with TBS, incubated with an avidin–alkaline phosphatase complex (ABC kit, Vectastain, Vector) for 30 min, and washed 3× with TBS. Alkaline phosphatase activity was visualized using Liquid Permanent Red Substrate-Chromogen (DAKO) for 15 min. After washing with water, slides were counterstained with Mayer’s hemalum diluted 1:1 in water for 10 s, blued under water, and mounted with Eukitt® (Sigma).

### Statistics

Two-tailed, unpaired Student’s *T*-test was used for pair-wise comparison of genotypes. Two-way ANOVA followed by Tukey's Test was used for comparisons among multiple groups. Analysis was performed using Microsoft Excel or GraphPad Prism. *P* < 0.05 was considered significant.

### µ-CT analysis

Calvaria, femurs, and vertebrae were analyzed by µCT scanning (µCT 40, Scanco Medical, Bassersdorf, Switzerland). Calvaria were scanned at 15 µm voxel size and quantification of calvarial porosity was performed on three-dimensional reconstructions using a custom-made threshold routine (Image J 1.42, National Institute of Health, USA). Femurs and vertebrae were scanned at 10 µm voxel size and analyzed using the evaluation software provided by the manufacturer (Scanco Medical, Bassersdorf, Switzerland).

### Co-culture

We isolated primary osteoblasts calvaria of 3-day-old mice as described in the manuscript in Primary cell isolation and culture. Briefly, 24 h after osteoblast isolation (8 × 10^3^ per well), osteoclast progenitor cells (2.5 × 10^5^ per well) from femur and tibia bone marrow of wild-type C57BL/6 were added to the osteoblast cultures. Cells were cultured in alpha-MEM supplemented with 100% FCS + 1% Pen/Strep + 10^−8^ mol·L^-1^ 1,25(OH)-dihydroxyvitamin D3 (Sigma) + 10^−7^ mol·L^-1^ dexamethasone (Sigma) + 20 ng·mL^-1^ M-CSF (Peprotech) in the presence or absence of 500 ng·mL^-1^ anti-PDGFRβ (Upstate) in 96-well plates. Media was changed every third day. At day 13, TRAP staining was performed to quantify the number of developing osteoclasts. We used a color threshold method with ImageJ to determine the percentage of TRAP-positive stained cells. Two independent experiments were performed in triplicates.

### Lipoprotein turnover studies

To analyze fatty acid and lipoprotein uptake, we performed a turnover experiment using radiolabeled tracers as previously described^31^. In brief, male mice (12 weeks old) were fasted for 4 h before receiving a gavage of 200 µL olive oil mixed with [9,10-^3^H(N)]-triolein (0.185 MBq per mouse) and 1.75 h after gavage, 150 µL of [Carboxyl-^14^C]-triolein (0.02 MBq per mouse) in recombinant lipoproteins was injected via the tail vein. Two hours later, the mouse was perfused transcardially with PBS (Gibco) containing 10 U·mL^-1^ heparin (Rotexmedica). To measure radioactivity, organs were solubilized in Solvable (PerkinElmer, 0.1 mL per 10 mg organ), 400 µL were counted in a scintillation fluid, and fatty acid as well as lipoprotein uptake was calculated as counts per minute (cpm) per mg organ.

## Electronic supplementary material

Supplementary Information
