# Supplementary material for: Lrp1 in osteoblasts controls osteoclast activity and protects against osteoporosis by limiting PDGF–RANKL signaling
Source: Bone Res. 2018 Feb 26;6:4. doi: 10.1038/s41413-017-0006-3 (PMC5826921; doi:10.1038/s41413-017-0006-3)
Supplement: Supplementary file 1 — Supplementary Information [file 41413_2017_6_MOESM1_ESM.pdf]

Lrp1 protects against osteoporosis by limiting PDGF-RANKL signaling in osteoblasts

Bartelt et al.

Supplementary Information

Bartelt et al. Supplementary Fig. 1

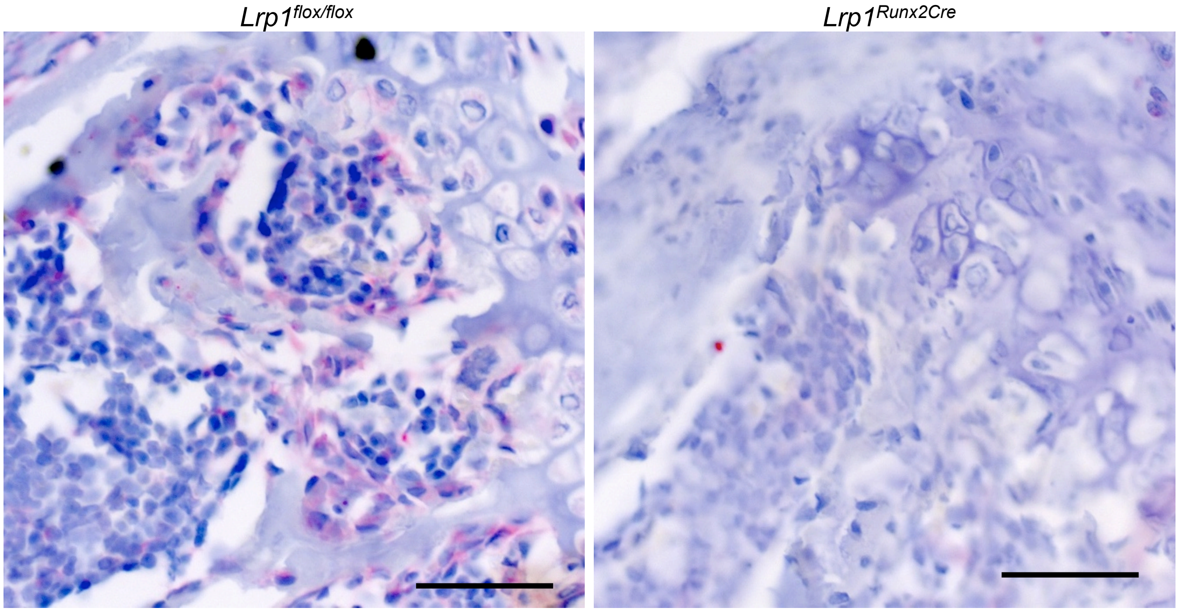

Supplementary Fig. 1 Bone Lrp1 immunohistochemistry. Metaphyseal trabecular bone from sagittal knee sections of 12 week-old female mice show strong Lrp1 protein expression in osteoblasts in wild-type mice, which was blunted *Lrp1*^{Runx2Cre} mice. Bar 50 µm.

Bartelt et al. Supplementary Fig. 2

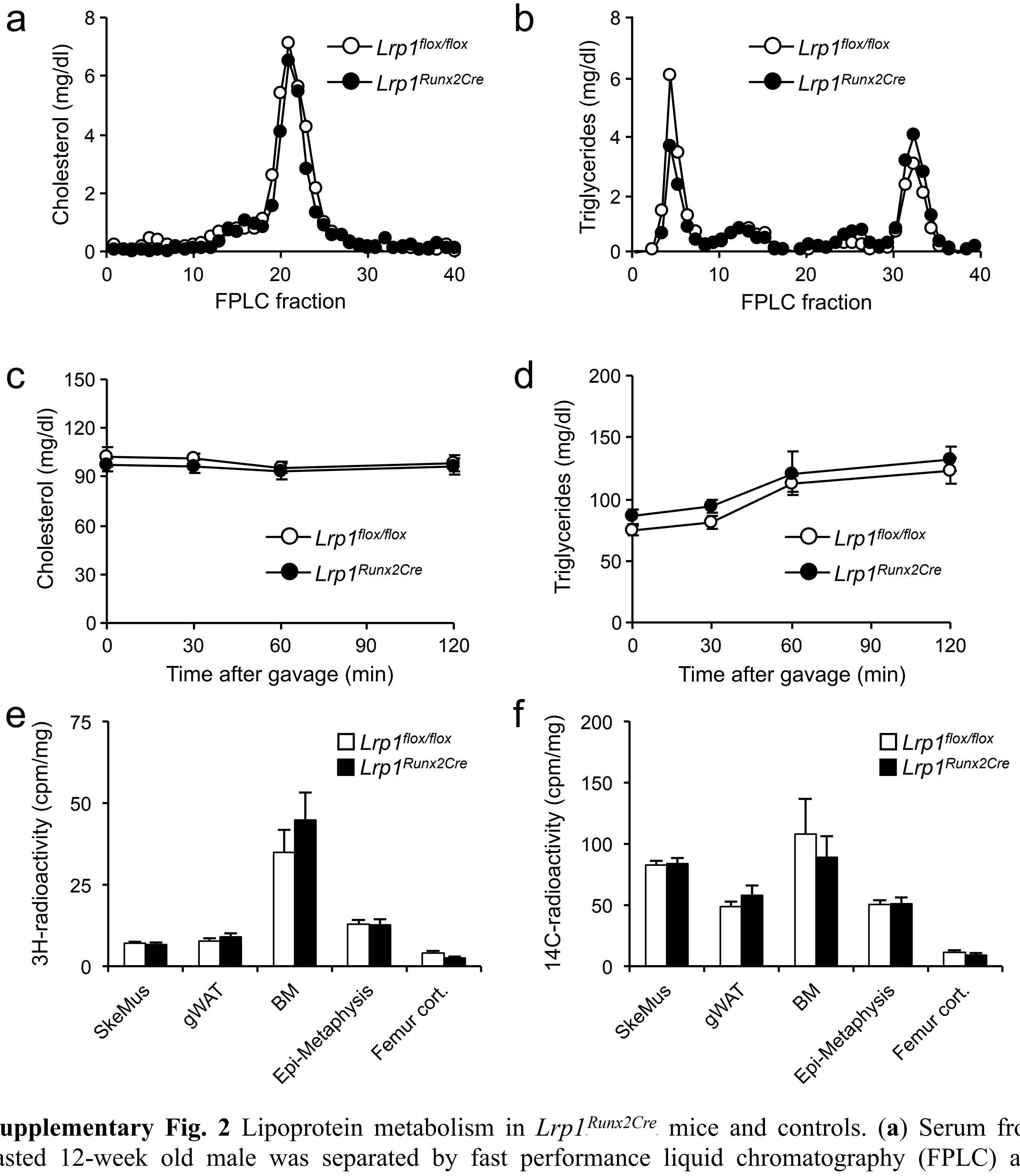

Supplementary Fig. 2 Lipoprotein metabolism in *Lrp1*^{Runx2Cre} mice and controls. (**a**) Serum from fasted 12-week old male was separated by fast performance liquid chromatography (FPLC) and cholesterol as well as (**b**) triglyceride concentrations were determined in each fraction. The lipoprotein profile remains unchanged in *Lrp1*^{Runx2Cre} mice compared to controls. (**c**) Also in a dynamic oral fat tolerance test with olive oil, cholesterol as well as (d) triglyceride levels remained unchanged in *Lrp1*^{Runx2Cre} mice. Means±s.e.m., n=6 per group. (e) Tissue uptake of fatty acids after ^{3}H-radiolabeled triolein oral gavage and (f) tissue recombinant lipoprotein uptake after i.v. injection in *Lrp1*^{Runx2Cre} mice and controls. Means±s.e.m., n=10 per group. SkeMus: Quadriceps femoris, gWAT: gonadal white adipose tissue, BM: Bone marrow.

Bartelt et al. Supplementary Fig. 3

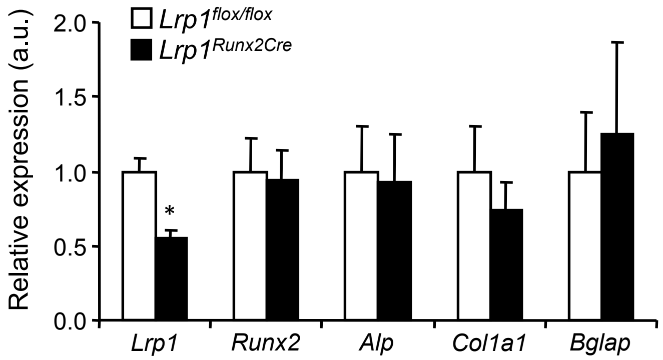

Supplementary Fig. 3 Primary osteoblasts isolated from *Lrp1*^{Runx2Cre} mice and controls. Expression of differentiation markers at d21 in osteoblasts from *Lrp1*^{Runx2Cre} mice compared to cells isolated from controls. Means±s.e.m. from 4 independent experiments in duplicates or triplicates (*, *P*<0.001 determined by unpaired, two-tailed T-test) (*Alpl, alkaline phosphatase; Bglap, Bone gamma-carboxyglutamate protein; Col1a1, collagen, type I, alpha 1; Lrp1, LDL receptor-related protein-1; Runx2, runt-related transcription factor 2*).

Bartelt et al. Supplementary Fig. 4

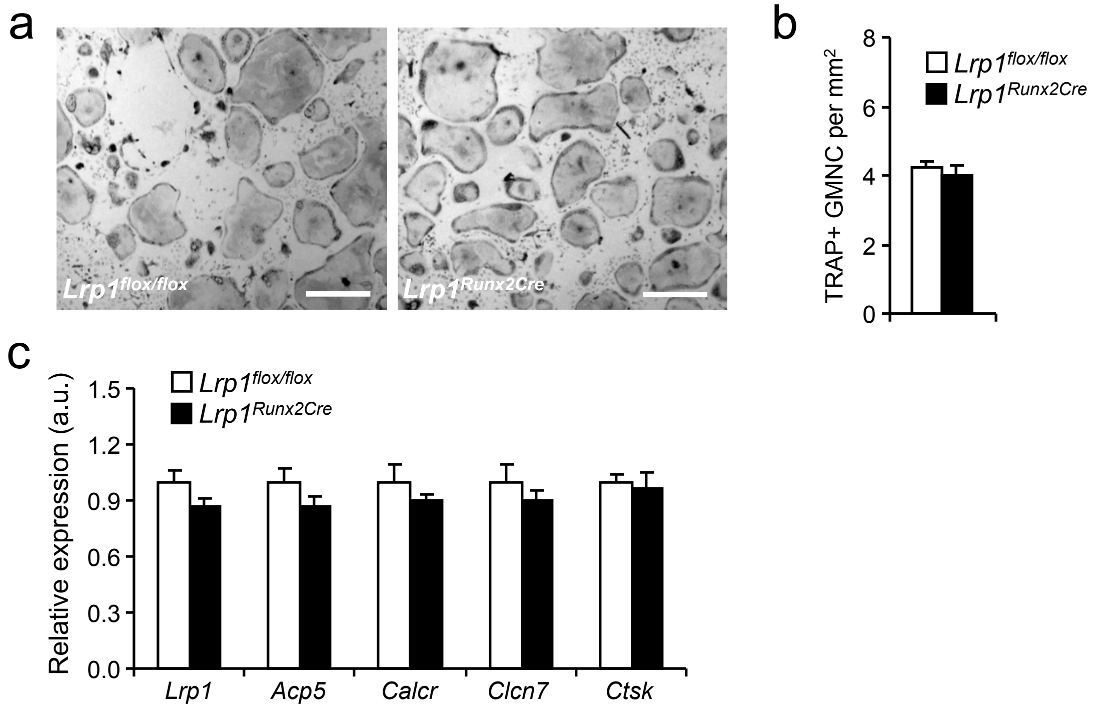

Supplementary Fig. 4 Primary osteoclasts isolated from *Lrp1*^{Runx2Cre} mice and controls. (**a**) Tartrate-resistant acid phosphatase (TRAP) staining in osteoclasts and (**b**) quantification of TRAP-positive giant multinucleated cells (GMNC) was unaltered in *Lrp1*^{Runx2Cre} mice compared to controls. Scale bar, 0.5 mm (**c**) Also the expression of genes important for osteoclast function remained unchanged in cells isolated from *Lrp1*P^{Runx2Cre}P mice compared to cells isolated from controls. Means±s.e.m. (*Acp5, acid phosphatase 5, tartrate resistant* (encoding TRAP)*; Calcr, calcitonin receptor; Clcn7, chloride channel 7; Ctsk, cathepsin K; Lrp1, LDL receptor-related protein-1*).

Bartelt et al. Supplementary Fig. 5

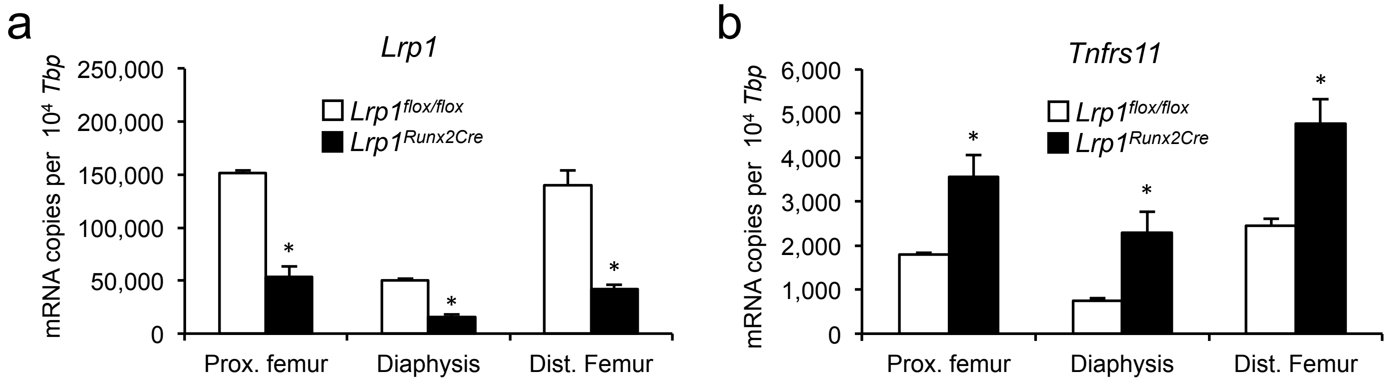

Supplementary Fig. 5 Gene expression mRNA level of (**a**) *Lrp1* and (**b**) *Tnfrs11* (Rankl) in the femoral compartments of 12-week old male $Lrp1^{Runx2Cre}$ mice and controls (n=3). Means±s.e.m (*, *P*<0.01 determined by unpaired, two-tailed T-test)

Bartelt et al. Supplementary Fig. 6

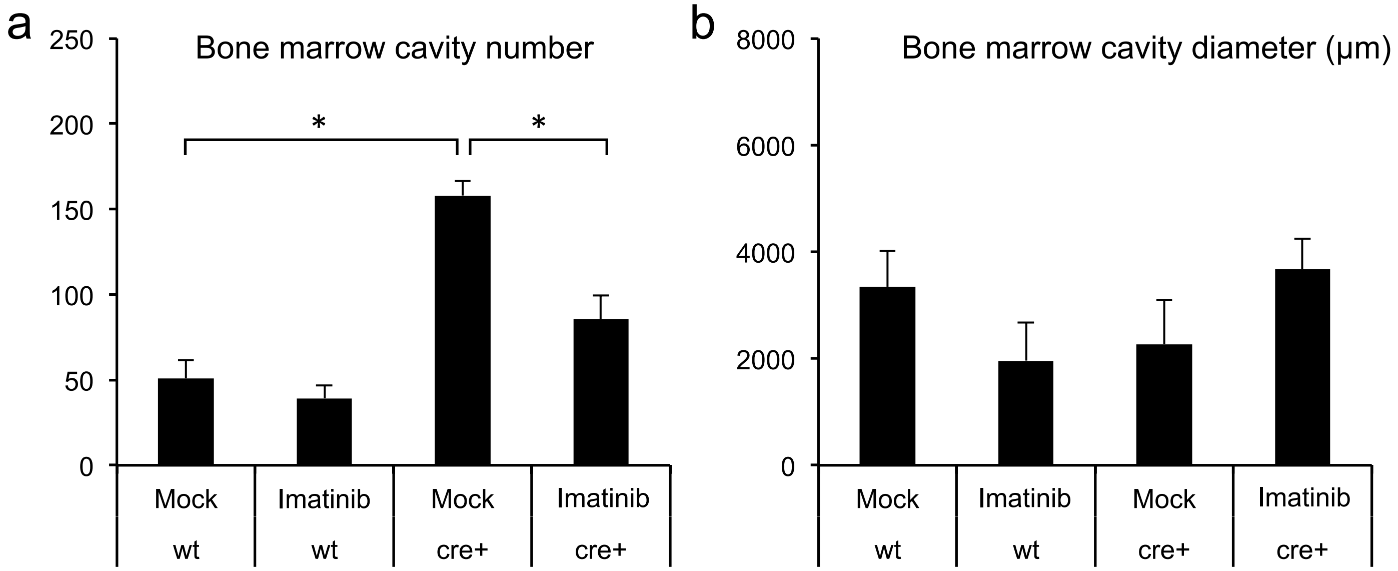

Supplementary Fig. 6 µCT analysis of calvaria from female 26 week old *Lrp1*^{Runx2Cre} mice (cre+) and littermates controls (WT) that received mock or Imatinib-containing diet (n=4-5). Means±s.e.m (*, *P*<0.05 determined by 2-way ANOVA followed by Tukey's test)
